# Molecular Phylogenetics and Mitochondrial Genomic Evolution in the Endemic Genus *Pielomastax* (Orthoptera: Eumastacoidea) in China

**DOI:** 10.3390/genes15101260

**Published:** 2024-09-27

**Authors:** Jun-Hui Lu, Keyao Zhang, Sheng-Quan Xu, Ying Ding

**Affiliations:** College of Life Sciences, Shaanxi Normal University, Xi’an 710119, Chinakeyaozh@163.com (K.Z.); xushengquan@snnu.edu.cn (S.-Q.X.)

**Keywords:** divergence time, Eumastacidae, *Pielomastax*, mitochondrial genome

## Abstract

Background/Objectives: The genus *Pielomastax* Chang (Orthoptera: Eumastacoidea, 1937) is endemic to China, which is mainly distributed in low- and medium-altitude areas in central and eastern China. However, there are relatively few molecular data studies on the genus *Pielomastax*. Methods: In this study, three species of the genus *Pielomastax* were collected from Hubei and Henan, China, namely *Pielomastax* sp., *Pielomastax shennongjiaensis* Wang (1995) and *Pielomastax tenuicerca* Hsia and Liu (1989). Both *Pielomastax* sp. and *Pielomastax shennongjiaensis* were collected from the Shennongjia area of Hubei, but they exhibit some differences in morphological characteristics. Results: We obtained the mitochondrial genome structures of the three species, which were similar to those of the published mitochondrial genome structures of species within Eumastacoidea with 37 typical mitochondrial genes, including 13 PCGs, 22 tRNAs, and 2 ribosomal RNAs. The results of the maximum likelihood (ML) tree and the Bayesian inference (BI) tree showed that the families Eumastacidae, Chorotypidae and Episactinae in Eumastacoidea are a monophyletic group, and Thericleinae and Episactinae are sister clades. The time-calibrated phylogeny results indicated that the divergence time between Thericleinae and Episactinae was 95.58 Ma (56.71–128.02 Ma). Conclusions: These phylogenetic tree results indicate that *Pielomastax* sp. and *Pielomastax shennongjiaensis* are the same species. And the time-calibrated phylogeny tree and the species distribution map of the genus *Pielomastax* indicate that the species of the genus *Pielomastax* spread from eastern to central China and diversified. These studies fill the gap in molecular data for the genus *Pielomastax* and the taxonomic status of Episactidae.

## 1. Introduction

The genus *Pielomastax* Chang (1937) belongs to Episactidae Burr (1899), which is only distributed in China in Asia, southern North America and Madagascar in Africa [1]. Almost all insects in the Episactidae family are wingless, which is a key factor contributing to their limited distribution range. There is controversy over the taxonomic and phylogenetic status of Episactidae. Studies on morphological characteristics suggest that Gomphomastacinae is closely related to Episactidae [2], but there is almost no research on the taxonomic status based on molecular data.

The genus *Pielomastax* [3] is endemic to China with a total of 14 species, mainly distributed in the central and eastern regions of China [4]. The genus *Pielomastax* has attracted extensive attention from researchers due to its unique distribution and morphological characteristics. The main characteristics of this genus are short, filamentous antennae and a lack of wings, and the main characteristics for the classification of this genus are the number of antennal segments and the shape of the male cerci and the supra-anal plate [4]. However, most of the research on this genus focuses on morphological characteristics, and relatively little research has been conducted on its molecular data.

The insect mitochondrial genome is a circular DNA molecule [5], usually about 15,000 to 18,000 base pairs in length. The insect mitochondrial genome has 37 genes, including 13 protein-coding genes, 22 tRNA genes, and 2 rRNA genes [6]. The mitochondrial genome plays an important role in the systematic evolutionary studies of insects [7] because its high variability and maternal inheritance mode make it an ideal marker for studying population genetics and evolutionary relationships. In addition, the structure and sequence characteristics of the insect mitochondrial genome also provide important information about mitochondrial function and adaptive evolution [8]. The arrangement order of genes and the variation of control regions can reveal how different species adapt to environmental pressures during evolution [9]. In recent years, with the advancement of sequencing technology, more and more insect mitochondrial genomes have been decoded, which can provide rich data support for in-depth studies of insect diversity and evolutionary history [10,11,12].

In this study, we collected three species from Hunan and Hubei, namely *Pielomastax* sp. (*P.* sp.), *Pielomastax shennongjiaensis* (*P. shennongjiaensis*) and *Pielomastax tenuicerca* (*P. tenuicerca*). The number of antennal segments and the shape of the male cerci and the supra-anal plate are the main taxonomic characteristics of *Pielomastax*. The morphological characteristics of the male cerci and supra-anal plate of *P.* sp. and *P. shennongjiaensis* are similar, but the antennae of *P. shennongjiaensis* have 9 segments, while the antennae of *Pielomastax* sp. have 10 segments. We determined the relationships of *P.* sp. and *P. shennongjiaensis*, the phylogenetic relationships within *Pielomastax* and the taxonomic status of Episactidae by sequencing the mitochondrial genomes of these three species and combining with the NCBI genome data from 12 species available online (8 Eumastacoidea species and 4 outgroups).

## 2. Materials and Methods

### 2.1. Specimen Collection, Morphological Data Acquisition and Mitogenome Sequencing

Three species of the genus Pielomastax were collected in this study, namely *P.* sp., *P. shennongjiaensis* and *P. tenuicerca* (Table 1). The specimens were collected in Hubei and Henan in 2023 and preserved in anhydrous ethanol. The specimens collected in this study were all adults and were numbered according to the collection location that were stored in the Animal Specimen Museum of Shaanxi Normal University. The specimens were observed using a VHX-6000 digital microscope (Keyence, Osaka, Japan), and the body size and antenna length of the specimens were measured using a Vernier caliper. Total genomic DNA was extracted from muscle tissues of the three species using the Dnasy Blood and Tissue Kit (Qiagen 69504, Hilden, Germany). Paired-end libraries with an insert length of 350 bp were prepared and sequenced on the Illumina NovaSeq 6000 platform (Illumina, San Diego, CA, USA) with a read length of 150 bp. The measured raw mitochondrial genome sequences were filtered by Fastp (Parameters: -w 8 -q 20 -u 30 -l 50) [13], and the filtered reads were assembled into draft mitochondrial genome sequences in MitoZ version 2.4 (De novo, clade set to “Arthropoda”) [14]. In addition, we manually checked the annotation information of mitochondria of the three species using genous11.1. The positions of the start and stop codons of protein coding genes (PGCs) of the three species were compared with the published mitochondrial genomes of two other species (*Pielomastax zhengi* and *Pielomastax soochowensis*) and adjusted according to the suggestions of Cameron (2014) [15]. The online tool Mitos2 (http://mitos2.bioinf.uni-leipzig.de/index.py, accessed on 28 May 2024) was used to predict transfer RNA genes of the three species [16].

MEGA [17] was used to analyze the nucleotide composition of the three mitochondrial genomes. OGDRAW [18] (https://chlorobox.mpimp-golm.mpg.de/OGDraw.html, accessed on 13 July 2024) was used to draw the mitochondrial gene maps of the three species. In order to determine whether the mitochondrial genomes of the three species had been remade, we compared the mitochondrial genomes of the three species in this study with the mitochondrial genomes of two closely related species (*Pielomastax zhengi* and *Pielomastax soochowensis*) published by NCBI to check for signs of insertion, deletion, reversal or recombination.

**Table 1:** List of specimens and sequences.

Family	Subfamily	Species	Accession Number	Elevation (m)	Location	Reference
Chorotypidae	Episactinae	*Pielomastax shennongjiaensis*	PQ325292	784.3	Shenlongjia, Hubei, China	This study
		*Pielomastax tenuicerca*	PQ325293	1201.4	Sanmenxia, Henan, China	This study
		*Pielomastax* sp.	PQ325294	1042.2	Shenlongjia, Hubei, China	This study
		*Pielomastax zhengi*	JF411955			[19]
		*Pielomastax soochowensis*	KM102728			[20]
	Erianthinae	*Chorotypus fenestratus*	KM657339			[21]
	Chininae	*China manfispoides*	OQ241410			[22]
Eumastacidae	Gomphomastacinae	*Phytomastax pentaspinula*	OQ241413			[22]
	Paramastacinae	*Paramastax nigra*	JX913772			[23]
Episactidae	Episactinae	*Erianthus versicolor*	OQ241411			[22]
Thericleidae	Thericleinae	*Pseudothericles compressifrons*	NC028061			[21]
Tetrigidae		*Tetrix japonica* *	NC018543			[24]
Tanaoceridae		*Tanaocerus koebelei* *	JX913774			[23]
Pyrgomorphidae		*Atractomorpha sinensis* *	EU263919			[25]
Acrididae		*Locusta migratoria* *	NC001712			[26]

Note: * Outgroup.

### 2.2. Phylogenetic Reconstruction

We searched the NCBI GenBank database for Eumastacoidea (‘txid92622 [Organism]’ as the keyword) and identified eight additional mitochondrial genome sequences (Table 1). The data in this study are from 4 families of Eumastacoidea [1], including 8 subfamilies. In addition, to analyze the phylogenetic relationships between the three species in this study and other species of Eumastacoidea, we selected four species in the suborder Caelifera as outgroups, namely, *Etrix japonica* Bolívar, 188 (Tetrigoidea), *Tanaocerus koebelei* Bruner, 1906 (Tanaoceroidea), *Atractomorpha sinensis* Bolívar, 1905 (Pyrgomorphoidea), and *Locusta migratoria* Linnaeus, 1758 (Acridoidea), respectively.

We extracted 13 PCGs sequences (without stop codons) of all species based on the mitochondrial genome annotation information of 3 species in this study and the mitochondrial genome annotation information of 12 species downloaded from NCBI. The protein-coding sequences of 13 mitochondrial PCGs from 15 species were aligned using MAFFT [27], and then the 13 PCGs were concatenated using Python script. IQTREE [28] was used to construct the maximum likelihood (ML) tree and MrBayes [29] was used for Bayesian inference (BI). For the ML analysis, 39 partitions were generated based on the positions of three codons of the 13 PCGs, and the best-fitting partitioning scheme and the best-substitution model that minimized the Akaike Information Criterion (AIC) (option-m TESTMERGE) score were subsequently selected using ModelFinder [30]. We selected the same best-fit partitioning scheme and partition-specific models in the BI analysis as in the ML analysis, and other parameters were selected for two simultaneous runs with four chains (three hot and one cold) for 2 million generations, sampling every 100 generations. Finally, Figtree was used to visualize the phylogenetic tree.

### 2.3. Estimating Divergence Time and Comparative Phylogenetic Analyses

In this study, a divergence time estimation analysis was performed using a relaxed clock log-normal model in BEAST version 2.7 [31] to estimate the divergence time of the Eumastacoidea. We used three estimates from the phylogeny reported by Song et al. (2015) that were constructed and time-calibrated using mitochondrial genome sequences, the crown age of Cealifera (224.44 Ma; 186.4–351.7 Ma), the crown age of all superfamilies within Cealifera except Tetrigoidea (196.97 Ma; 163.8–349.9 Ma), and the divergence time between *Erianthus versicolor* Brunner, 1898 and *Chorotypus fenestratus* Serville, 1838 (123.52 Ma; 61.7–205.3 Ma), respectively [21]. The Yule model was used as the tree prior, the best partition schemes and evolution models determined by ModelFinder based on AIC, and the Markov chain was run for 20 million generations with sampling every 2000 generations. Tracer version 1.7 [32] was used to determine convergence after which the top 10% were discarded as burn-in. And FigTree was used to visualize maximum clade credibility tree.

## 3. Results

### 3.1. Mitochondrial Genome Assembly and Annotation

**Figure 1:**
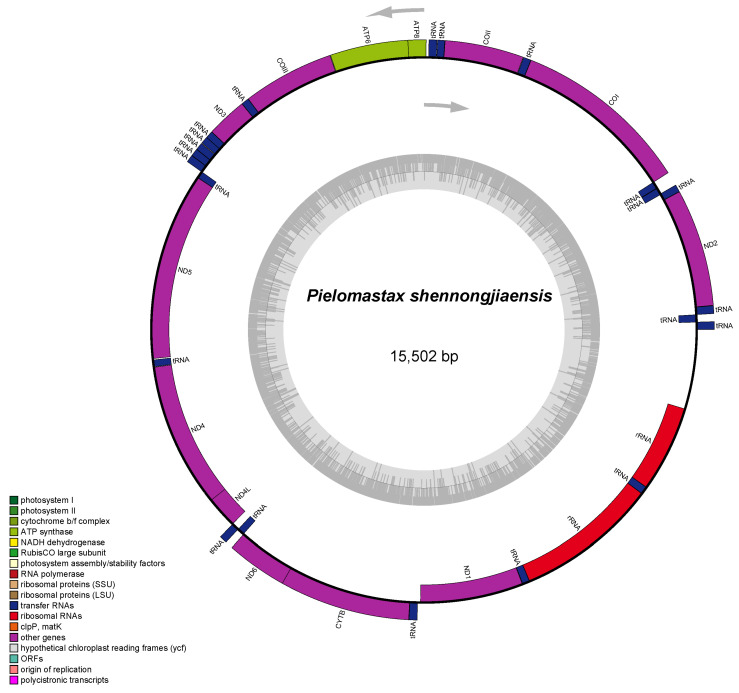
Mitochondrial genome map of *P. shennongjiaensis*.

In this study, three complete mitochondrial genomes were assembled with approximately 4 Gb of paired-end sequencing reads per species, ranging from 15,502 (*P. shennongjiaensis*) (Figure 1) to 15,534 (*P.* sp.) (Table S1, Figure S1). All three assembled mitochondrial genomes exhibit a similar nucleotide composition, with a bias toward AT nucleotides. The GC content of the three mitochondrial genomes was low, with *P.* sp. and *P. shennongjiaensis* having similar GC contents of 30.30% and 30.20%, respectively. *P. tenuicerca* (Figure S2) had the lowest GC content of 28.40%. The structures of the three complete mitochondrial genomes were identical to the previously published Eumastacoidea genome with 37 typical mitochondrial genes, including 13 PCGs, 22 tRNAs, and 2 ribosomal RNAs. In terms of gene arrangement, the three mitochondrial genomes maintained the same gene order and directionality as typical insect mitochondrial genomes. By analyzing the non-coding regions of the mitochondrial genome, we found some conserved sequences, especially the D-loop region, whose length and sequence composition were different in the three species. This region may be important in species-specific regulatory mechanisms.

### 3.2. Phylogenetic Reconstruction

**Figure 2:**
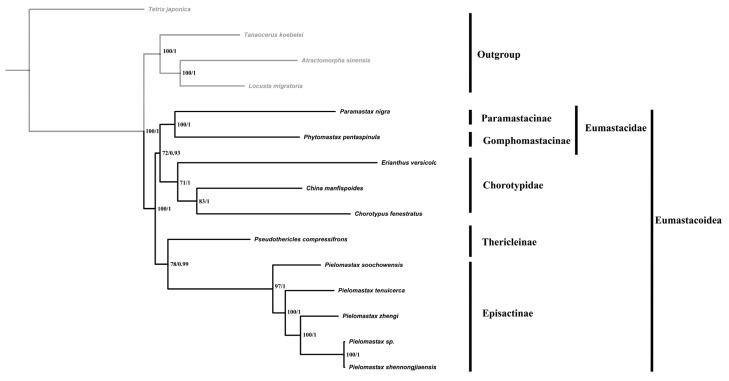
The phylogeny of Eumastacoidea reconstructed by maximum likelihood and a Bayesian analysis of 13 protein-coding genes linked to mitochondria. The numbers on the nodes are bootstrap support values and posterior probability values.

To determine the taxonomic status of *Pielomastax* within Episactinae, we constructed ML and BI trees (Figure 2) using a concatenated dataset of 13 mitochondrial PCGs. The results of the phylogenetic tree show that most of the nodes have high bootstrap support and BPP. Moreover, the phylogenetic tree shows that the superfamily Eumastacoidea and the families Eumastacidae, Chorotypidae and Episactinae are a monophyletic group. Thericleinae and Episactinae are sister clades, while Eumastacida and Chorotypidae are sister clades. *P. shennongjiaensis* and *P.* sp. are closely related, indicating that the two species are the same species.

### 3.3. Divergence Time Estimation and PGLS Analysis

**Figure 3:**
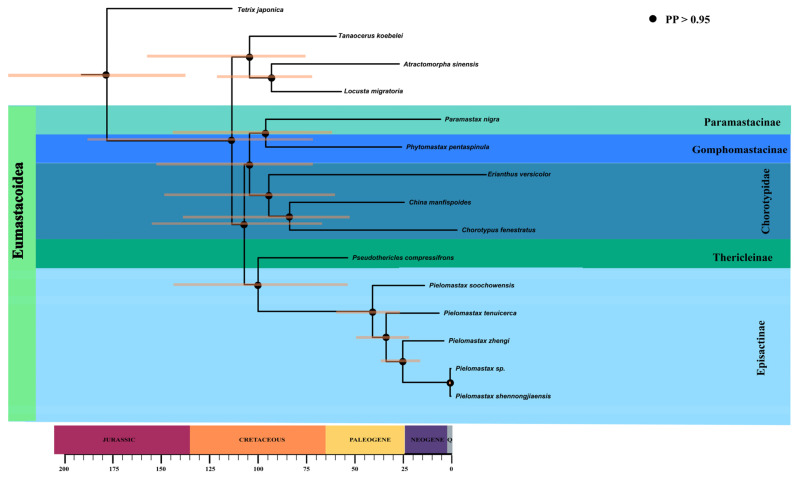
Time-calibrated phylogeny of Eumastacoidea for 13 protein-coding genes in 13 mitochondria with BEAST analysis. Black circles indicate posterior probability (PP) values over 95. Light orange bars represent 95% confidence intervals for node age.

The time-calibrated phylogeny we obtained using BEAST has the same topology as the ML and BI trees, and the posterior probability values of all nodes are greater than 0.95 (Figure 3). The estimated divergence time between the families Thericleidae and Episactidae was 95.58 Ma (56.71–128.02 Ma). And the estimated divergence time of the families Thericleidae and Episactidae and Chorotypidae was 118.64 Ma (74.62–167.78 Ma).

The divergence time between *Pielomastax soochowensis* and other species of *Pielomastax* was approximately 48.05 Ma (26.39–75.40 Ma). The divergence time between *P. shennongjiaensis* and *P.* sp. was 0.55 Ma (0.25–0.92 Ma). This indicates that the two species diverged very recently on the geological time scale and are most likely the same species.

## 4. Discussion

### 4.1. Insect Mitochondrial Genome

Insect mitochondrial genome plays an important role in biological research that is widely used in many fields such as molecular evolution, phylogenetics, population genetics and ecology [33,34]. In the mitochondrial genome, the COI gene is widely used in DNA barcoding technology for species identification and classification [35]. A study successfully identified multiple Lepidoptera species by analyzing the COI gene sequence of Lepidoptera, especially between populations that are difficult to distinguish in morphology [36]. Insect mitochondrial genomes have also played a key role in studying adaptive evolution [37]. Studies have found that mitochondrial genes in the Pteromalidae and Eulophidae families play an evolutionary role in adapting to plateau environments [38]. By comparing and analyzing the mitochondrial genomes of 11 plateau species and 18 lowland species, it was found that genes such as ATP6, ATP8, COX1, COX3, and CYTB were positively selected during plateau adaptation.

The A+T content of the mitochondrial genomes of the three species in this study are high, which is consistent with the characteristics of the mitochondrial genomes of most insects [5]. Our study found that the A+T content of these species are about 70%, which is a low level among Orthoptera insects. After comparison with other Orthoptera insects (such as locusts and katydids), it was found that their A+T content is usually between 75% and 85% [39,40]. This shows that the mitochondrial genome of this species has a certain degree of conservation in nucleotide composition, but there are still slight differences between species, which may be related to its ecological adaptability.

The most common type of mitochondrial genome rearrangement in Orthoptera is a change in the position of tRNA genes [40]. In some katydids (Tettigoniidae), the position of tRNA genes has been found to be different from that of other Orthoptera [41]. Some studies have found that tRNA rearrangements may be caused by slippage or unequal crossing-over in the genome replication mechanism [42,43]. However, we did not find obvious gene recombination events in the mitochondrial genomes of this study. Although mitochondrial DNA recombination has been reported in some invertebrates, it seems to be relatively rare in the Orthoptera in this study. This result is consistent with previous studies on other Orthoptera species, indicating that the mitochondrial genome of Orthoptera has maintained a relatively stable structure during evolution, which may contribute to the conservation of its function.

Insect mitochondrial genomes are widely used in phylogenetic studies, especially in studying the relationship and evolutionary history between species [5]. Some studies have used mitochondrial genomes to analyze the phylogenetic relationships between Lepidoptera at different taxonomic levels to determine the taxonomic status of Lepidoptera [44]. Phylogenetic relationships of 70 mitochondrial genes of Membracoidea revealed that all subfamilies (sensu lato) were recovered as monophyletic groups [45].

### 4.2. Phylogenetic Position of Pielomastax in Eumastacoidea

The genus *Pielomastax* was originally placed in the family Eumastacoidea based on the morphological characteristics of the male genitalia [4]. However, its phylogenetic position remains controversial, and molecular phylogenetic studies have not yet got a precise conclusion [46]. Studies on morphological characteristics suggest that Gomphomastacinae is closely related to Episactidae (Amedegnato 1993). And Song et al. (2015) showed that Episactidae and Thericleidae were sister clades [21], which is consistent with the results of this study. The African Thericleidae or Episactidae clustered together, Gomphomastacinae clustered with Paramastacinae, and the Asian Chorotypidae clustered with Gomphomastacinae and Paramastacinae.

### 4.3. Diversification within Episactinae

**Figure 4:**
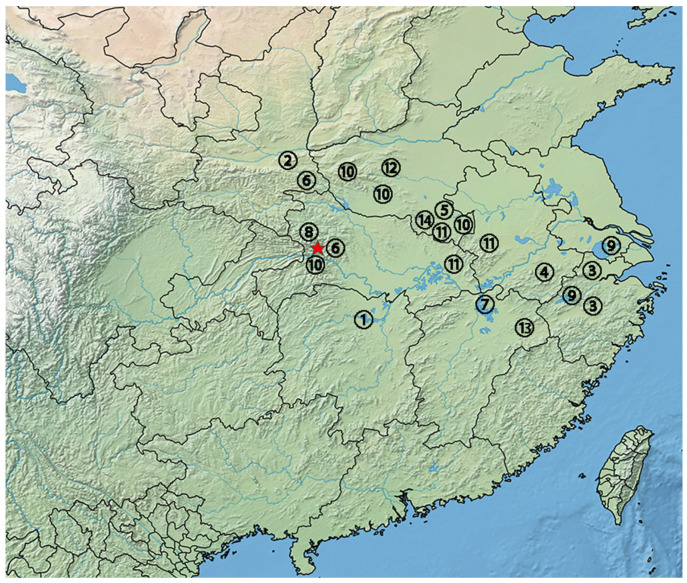
Distributed localities of the *Pielomastax* spp. in China. The serial numbers in the circles represent published species, respectively (1 *Pielomastax acuticerca*, 2 *Pielomastax cladopygidium*, 3 *Pielomastax cylindrocerca,* 4 *Pielomastax guliujiangensis*, 5 *Pielomastax lobata*, 6 *Pielomastax obtusidentata*, 7 *Pielomastax octavii*, 8 *P. shennongjiaensis*, 9 *Pielomastax soochowensis*, 10 *P. tenuicerca*, 11 *Pielomastax tridentata*, 12 *Pielomastax varidentata*, 13 *Pielomastax wuyishanensis*, 14 *Pielomastax zhengi*). The red five-pointed star represents *P.* sp.

Episactinae is mainly distributed in eastern and western China, Yunnan and Inner Mongolia in Asia. The genus *Pielomastax* is endemic to China and is only distributed in eastern and western China (Figure 4). During the Miocene, the Tibetan Plateau rose significantly that led to the differentiation of ecosystems and species in eastern and western China [47]. The cold and dry environment in the west led to the specialization of mammals and birds, while the humid climate in the east favored the diversification of temperate and subtropical plants. The Tibetan Plateau continued to rise during the Pleistocene [48], and the Pleistocene glaciation caused some species to migrate to more suitable environments.

The divergence time between *Pielomastax soochowensis* and other species of *Pielomastax* was approximately 48.05 Ma (26.39–75.40 Ma). *Pielomastax soochowensis* is located in the easternmost part of China, Suzhou, Jiangsu (Figure 4). The divergence time of *P. tenuicerca* from three species (*P. shennongjiaensis*, *Pielomastax zhengi* and *P.* sp.) is 39.16 Ma (20.87–61.68 Ma). *P. tenuicerca* is distributed in Henan and Hubei, which suggests that *P. tenuicerca* has spread during its evolution. *Pielomastax zhengi* is only distributed in Henan which is west of the easternmost distribution point of *P. tenuicerca*. The divergence time between *P. shennongjiaensis* and *P.* sp. was 0.55 Ma (0.25–0.92 Ma). *P. nnongjiaensis* and *P.* sp. are located in eastern China, and this result indicates that species of the genus *Pielomastax* spread from eastern China to central China.

## 5. Conclusions

This study assembled mitochondrial genome maps of three species of the genus *Pielomastax*, namely *P.* sp., *P. shennongjiaensis* and *P. tenuicerca*. The phylogenetic trees of ML and BI and a time-calibrated phylogeny showed that *P.* sp. and *P. shennongjiaensis* are the same species. This result showed that it is unreliable to distinguish the species of the genus *Pielomastax* only by the number of antennae segments. In addition, the results of the phylogenetic tree show that Thericleinae and Episactinae are sister clades, and the divergence time of Thericleinae and Episactinae was 95.58 Ma (56.71–128.02 Ma). These findings not only provide new insights into the classification of *Pielomastax* species, but also help to deepen our understanding of the phylogenetic relationships and evolutionary history of the Episactidae family.

## Data Availability

The mitochondrial genome data are publicly available on National Center for Biotechnology Information (PQ325292, PQ325293, PQ325294).

## Supplementary Materials

The following supporting information can be downloaded at: https://www.mdpi.com/article/10.3390/genes15101260/s1, Figure S1: Mitochondrial genome map of *Pielomastax* sp. Figure S2: Mitochondrial genome map of *Pielomastax tenuicerca*. Table S1: Nucleotide composition of five mitochondrial genomes.
