# Supplementary material for: Molecular Phylogenetics and Mitochondrial Genomic Evolution in the Endemic Genus Pielomastax (Orthoptera: Eumastacoidea) in China
Source: Genes (Basel). 2024 Sep 27;15(10):1260. doi: 10.3390/genes15101260 (PMC11507007; doi:10.3390/genes15101260)
Supplement: Supplementary file 1 [file genes-15-01260-s001.zip › Figure S1.pdf]

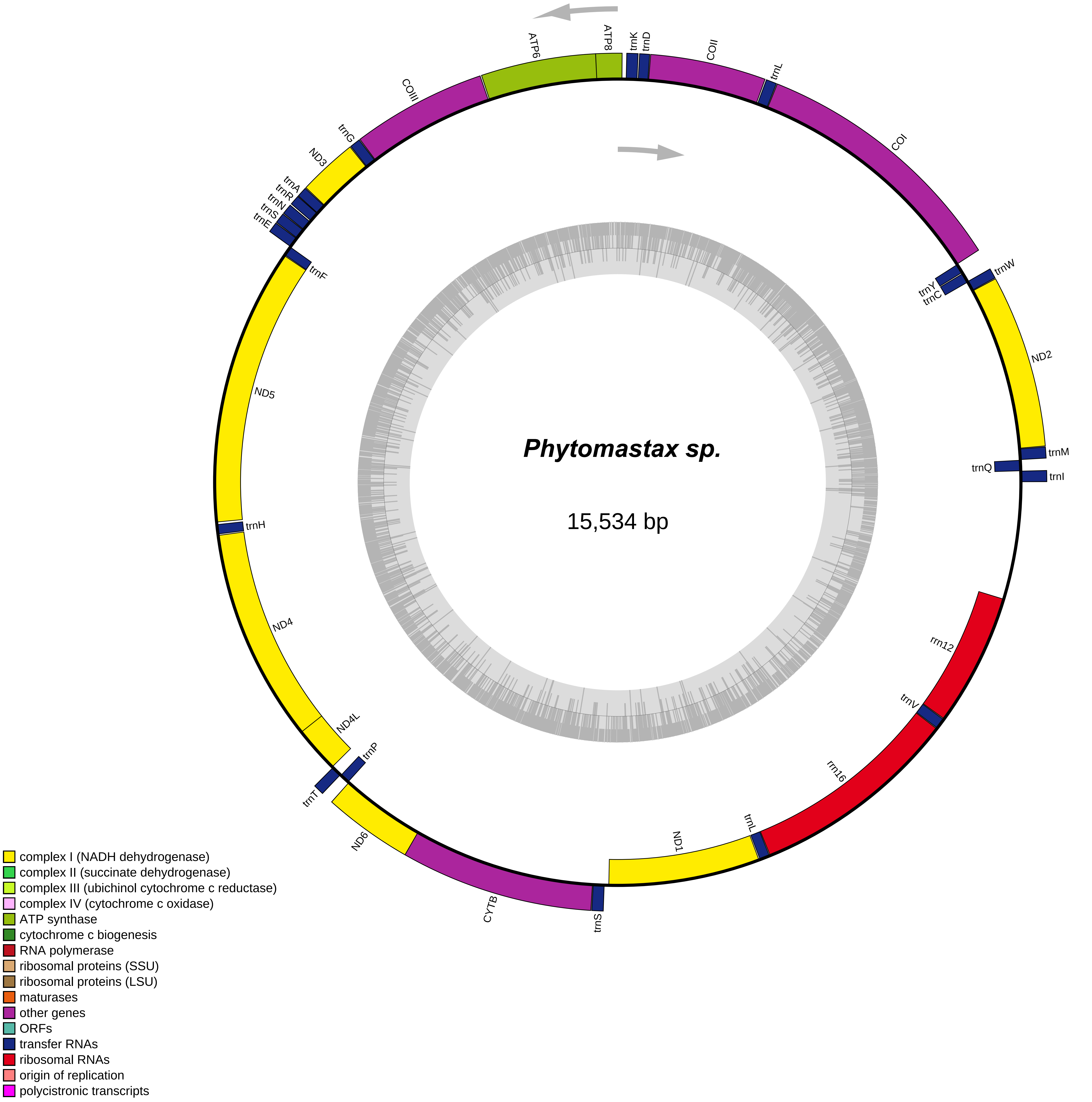
Phytomastax sp.
15,534 bp
ATP8
trnK
trnD
COII
trnL
COI
trnY
trnC
trnW
ND2
trnM
trnQ
trnI
rrn12
trnV
rrn16
trnL
ND1
trnS
CYTB
ND6
trnT
trnP
ND4L
ND4
trnH
ND5
trnF
trnE
trnS
trnN
trnR
trnA
ND3
trnG
COIII
ATP6
complex I (NADH dehydrogenase)
complex II (succinate dehydrogenase)
complex III (ubichinol cytochrome c reductase)
complex IV (cytochrome c oxidase)
ATP synthase
cytochrome c biogenesis
RNA polymerase
ribosomal proteins (SSU)
ribosomal proteins (LSU)
maturases
other genes
ORFs
transfer RNAs
ribosomal RNAs
origin of replication
polycistronic transcripts
