# Supplementary figures and images for: Molecular Phylogenetics and Mitochondrial Genomic Evolution in the Endemic Genus Pielomastax (Orthoptera: Eumastacoidea) in China
Source: Genes (Basel). 2024 Sep 27;15(10):1260. doi: 10.3390/genes15101260 (PMC11507007; doi:10.3390/genes15101260)

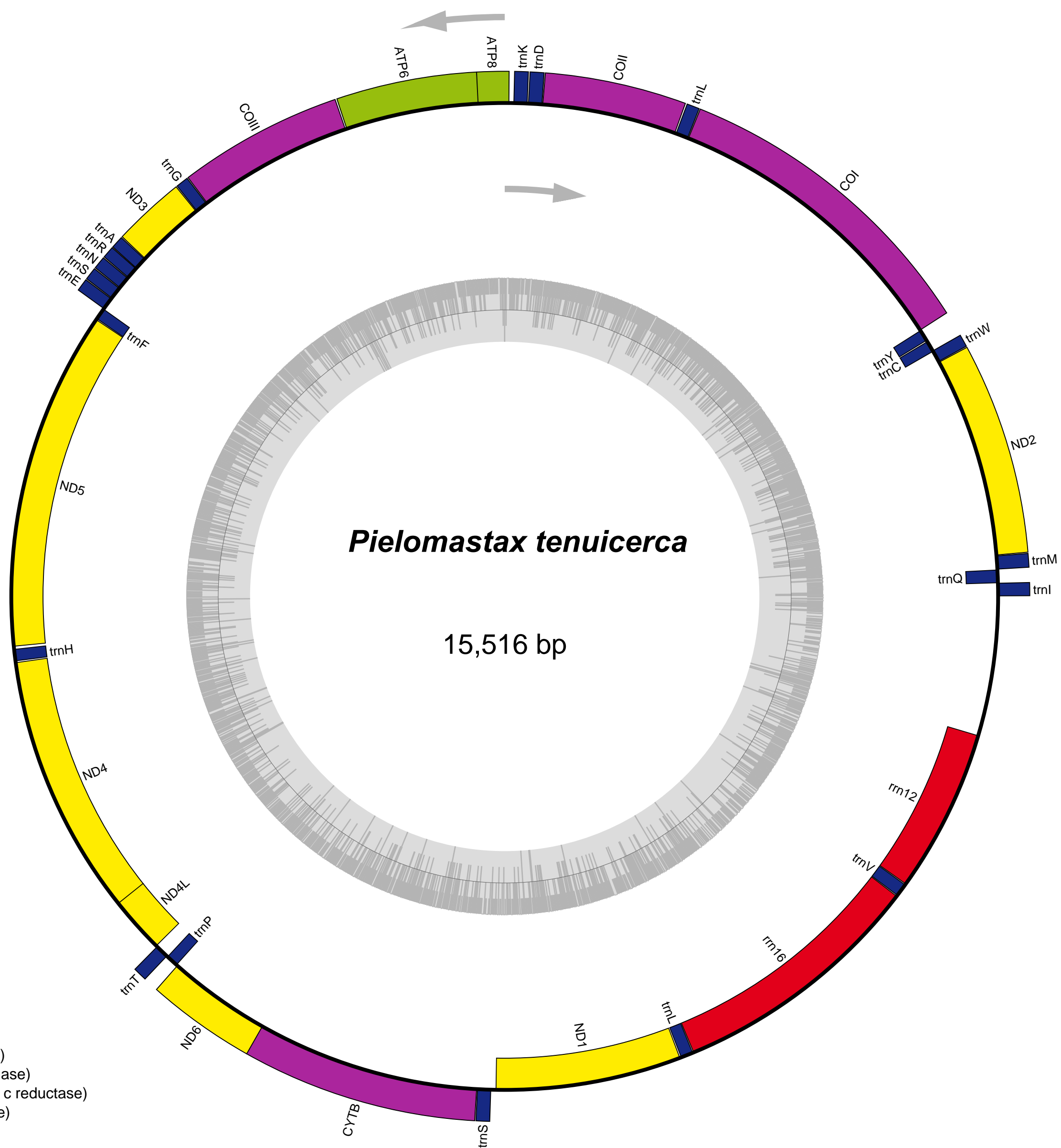

Supplement: Supplementary file 1 [file genes-15-01260-s001.zip › Figure S2.pdf]
